# Leisure-related cognitive reserve and postoperative cognitive outcomes after brain tumor surgery

**DOI:** 10.1093/noajnl/vdag175

**Published:** 2026-07-27

**Authors:** Masashi Kinoshita, Kota Ebina, Chiaki Domoto, Riho Nakajima, Mitsutoshi Nakada, Mie Matsui

**Affiliations:** Department of Neurosurgery, Faculty of Medicine, Institute of Medical, Pharmaceutical and Health Sciences, Kanazawa University, Kanazawa, Ishikawa, Japan; Smart-Aging Research Center, Tohoku University, Sendai, Miyagi, Japan; Institute of Liberal Arts and Science, Kanazawa University, Kanazawa, Ishikawa, Japan; Department of Occupational Therapy, Faculty of Health Science, Institute of Medical, Pharmaceutical and Health Sciences, Kanazawa University, Kanazawa, Ishikawa, Japan; Department of Neurosurgery, Faculty of Medicine, Institute of Medical, Pharmaceutical and Health Sciences, Kanazawa University, Kanazawa, Ishikawa, Japan; Sapiens Life Sciences, Evolution and Medical Research Center, Kanazawa University, Kanazawa, Ishikawa, Japan; Institute of Liberal Arts and Science, Kanazawa University, Kanazawa, Ishikawa, Japan

**Keywords:** brain tumor, cognitive function, cognitive reserve, leisure activity, white matter

## Abstract

**Background:**

Cognitive reserve (CR) is increasingly recognized as a factor associated with cognitive outcomes in neurological diseases; however, its relevance in patients undergoing brain tumor surgery remains incompletely understood. This study aimed to investigate the relationships between CR, particularly leisure-related CR, postoperative cognitive outcomes, and white matter tract disconnection.

**Methods:**

Forty patients in the chronic postoperative phase following supratentorial brain tumor resection were evaluated using the Mini-Mental State Examination (MMSE) and the Japanese Adult Reading Test (JART). Cognitive reserve was assessed using the Cognitive Reserve Index (CRI), including education, occupational attainment, and leisure subdomains. Postoperative structural magnetic resonance imaging was analyzed using an atlas-based disconnection approach (BCBtoolkit and Tractotron) to quantify tract-specific white matter disconnection. Associations among cognitive reserve, cognitive performance, and white matter disconnection were examined using correlation analyses and multivariable regression with stepwise variable selection.

**Results:**

MMSE and JART scores were positively correlated after covariate adjustment. Among CRI subdomains, only leisure-related CR was significantly associated with both MMSE and JART scores. Disconnection of the left inferior fronto-occipital fasciculus and the right posterior superior longitudinal fasciculus was associated with lower JART scores. Higher leisure-related CR was associated with relatively preserved cognitive performance in the presence of white matter tract disconnection.

**Conclusions:**

Leisure-related cognitive reserve was associated with postoperative cognitive outcomes in the context of white matter disconnection after brain tumor surgery. These findings suggest that individual differences in cognitive reserve may contribute to variability in postoperative cognitive outcomes and be informative for understanding cognitive vulnerability following tumor resection.

## Graphical Abstract

**Graphical Abstract:**
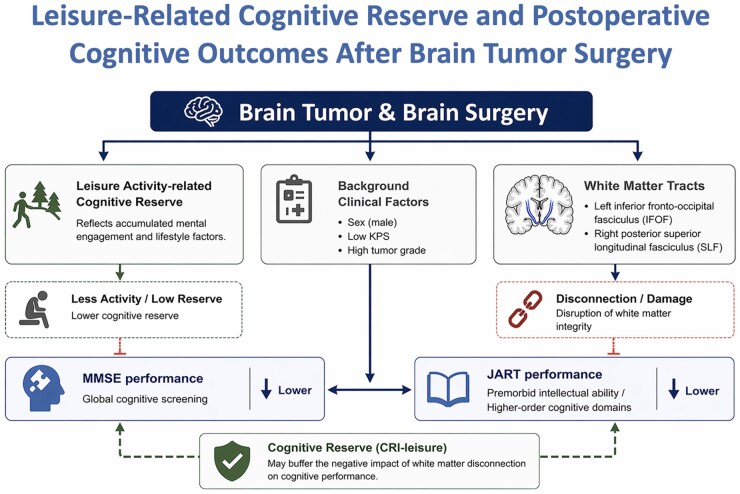

Key Points

- Leisure cognitive reserve was associated with cognitive outcomes after surgery.
- Disconnection of specific white matter tracts was associated with complex cognition.
- Cognitive vulnerability varied across individuals beyond anatomical factors alone.

Importance of the Study

Cognitive outcomes after brain tumor surgery show substantial interindividual variability that is not fully explained by lesion location or extent of resection alone. This study highlights the relevance of cognitive reserve, particularly leisure-related cognitive reserve, in understanding this variability. By integrating postoperative white matter tract disconnection analysis with cognitive reserve assessment, we show that leisure-related cognitive reserve is associated with postoperative cognitive outcomes, whereas education- and occupation-related reserve are not. In addition, disconnection of specific association tracts was associated with performance on a crystallized cognitive measure, underscoring tract-level vulnerability. Together, these findings provide a clinically relevant framework for understanding postoperative cognitive vulnerability and support consideration of cognitive reserve as a complementary factor alongside anatomical information when interpreting cognitive outcomes after brain tumor surgery.

Cognitive reserve (CR) refers to an individual’s capacity to preserve cognitive performance despite cerebral pathology or age-related changes and has been proposed as a key factor underlying interindividual variability in cognitive outcomes.^1^^,^^2^ Originating from observations in Alzheimer’s disease—where some individuals with significant neuropathological burden exhibited minimal clinical symptoms—CR has been conceptualized as a potential buffer against cognitive decline mediated by both structural and functional brain adaptations.^1^^,^^3^ This concept has been supported by numerous studies highlighting the association of higher levels of education, occupational attainment, and engagement in cognitively stimulating leisure activities with better cognitive health.^4^^,^^5^

Patients undergoing brain tumor surgery represent a population in which the concept of cognitive reserve may be particularly relevant yet remains underexplored. Unlike neurodegenerative diseases, brain tumors frequently cause acute disruption of cortical and subcortical structures, including white matter tracts critical for cognitive and language functions.^6^^,^^7^ Recent research has increasingly focused on the preservation of higher-order cognitive functions, in addition to motor and language outcomes, after brain tumor surgery.^8^ However, limited research exists on how individual differences in CR may influence cognitive outcomes following tumor resection.

Recent functional and structural imaging studies have provided insights into the neural substrates of CR, implicating large-scale networks such as the frontoparietal control system and temporo-limbic structures.^9^ Moreover, diffusion tractography and voxel-based lesion-symptom mapping have identified key white matter tracts, including the inferior fronto-occipital fasciculus (IFOF), superior longitudinal fasciculus (SLF), and cingulum bundle, as integral to cognitive integration.^10^^,^^11^ Disrupting these tracts during surgery may result in significant cognitive deficits, including semantic processing, working memory, and attention.^12^ However, interindividual differences in CR may influence or shape these associations.

Patients undergoing brain tumor surgery show substantial variability in postoperative cognitive outcomes, even when structural brain damage appears comparable. The factors underlying this heterogeneity remain poorly understood. Accordingly, the aim of this study was to examine the relationships among cognitive reserve, its subdomains, white matter disconnection, and postoperative cognitive outcomes in patients undergoing brain tumor surgery. We addressed the following hypotheses: (1) Higher cognitive reserve would be associated with better postoperative cognitive outcomes. (2) Different subdomains of cognitive reserve (education, occupation, and leisure activities) would show differential associations with postoperative cognitive outcomes. (3) Cognitive reserve would be related to cognitive outcomes when considered alongside structural network disruption following brain tumor surgery. Together, these hypotheses guided an integrated and exploratory analysis of cognitive reserve, white matter disconnection, and postoperative cognitive outcomes.

## Methods

### Participants

In this study, we recruited 40 patients who had undergone surgical resection of supratentorial brain tumors at Kanazawa University Hospital. All tumors were primarily located within the brain parenchyma or lateral ventricles, and only cases requiring surgical resection of brain tissue were included. Eligible patients were those who were at least 3 months postoperatively, had stable clinical conditions, and maintained a Karnofsky Performance Status (KPS) score of ≥70%, a cutoff applied to ensure reliable neuropsychological assessment and to minimize confounding effects of poor general condition on cognitive performance. The KPS is a clinician-rated scale ranging from 0 to 100 that reflects a patient’s functional status and ability to perform daily activities.^13^ Neuropsychological assessments were performed during the chronic postoperative phase, at a mean of 30.8 months after surgery (range, 3-296 months). All participants were clinically stable, with no signs of tumor recurrence and sufficient cognitive capacity to complete neuropsychological testing. The study protocol was approved by the Kanazawa University Medical Ethics Review Committee (approval no. 2897) and conducted in accordance with the principles of the Declaration of Helsinki. All participants were in the chronic postoperative phase, with no signs of tumor recurrence and sufficient cognitive capacity to complete neuropsychological testing. All patients provided written informed consent under the institutional ethics guidelines.

### Neuropsychological Assessment

Cognitive function was assessed using the Mini-Mental State Examination (MMSE) as a global cognitive screening tool and the Japanese Adult Reading Test (JART) as an index of premorbid intelligence, which has been shown in previous studies to be associated with higher-order cognitive functions.^14^ The JART assesses the reading ability of irregular kanji words and is widely used to estimate premorbid intellectual function reflecting crystallized verbal ability; its scores are standardized to a mean of 100 with a standard deviation of 15, similar to IQ measures.^14^ CR was quantified using the Japanese version of the Cognitive Reserve Index (CRI), which was specifically developed for the Japanese population based on the original CRI questionnaire created in Italy.^15^ This index comprises the following 3 subdomains: education (CRI-education), occupation (CRI-work), and leisure activities (CRI-leisure).^16^ A composite total score (CRI-total) was also calculated. Although the CRI questionnaire was administered postoperatively, it is designed to capture lifelong exposure to education, occupational attainment, and leisure activities. Accordingly, CRI scores were conceptualized as proxies of premorbid cognitive reserve rather than postoperative cognitive status.

### MRI Acquisition and Disconnection Analysis

Postoperative 3-dimensional T1-weighted MRI scans were obtained for each patient at the time of neuropsychological assessment to acquire structural brain data (Supplementary Figure S1). In all cases, gadolinium-enhanced T1-weighted and T2/FLAIR images were also acquired to confirm the absence of tumor recurrence. Images were normalized to MNI152 standard space using SPM12 (http://fil.ion.ucl.ac.uk/spm/). Surgical cavities were manually segmented and designated as regions of interest (ROIs) using MRIcron (https://www.nitrc.org/projects/mricron). Disconnection measures were based on resection cavity–derived ROIs on postoperative MRI. Although these ROIs primarily reflect surgical effects, their extent is constrained by tumor characteristics; thus, disconnection was interpreted as a composite postoperative structural measure. These ROIs were registered to the BCBtoolkit framework (http://toolkit.bcblab.com/), ^17^ and disconnection analyses were conducted using Tractotron^18^ to calculate disconnection ratios for major white matter tracts. Specifically, for 68 white matter tracts defined in the standard brain, the maximum proportion of each tract intersected by the resection cavity ROI was calculated. This atlas-based disconnection approach was selected because it enables standardized estimation of tract-level vulnerability using postoperative structural MRI alone, which is particularly suitable for retrospective cohorts in which preoperative diffusion MRI is unavailable or heterogeneous.

### Statistical Analysis

Statistical analyses proceeded in 4 steps: (1) descriptive statistics; (2) correlation analyses between cognitive reserve measures and cognitive outcomes; (3) multivariable linear regression analyses adjusting for clinical covariates; and (4) sensitivity analyses. Descriptive statistics were used to summarize demographic and clinical characteristics, and Pearson correlation analyses were performed to examine associations between MMSE/JART scores and each CRI subdomain.

Multivariable linear regression models were constructed, adjusting for age, sex, KPS, World Health Organization (WHO) tumor grade, history of radiotherapy, and postoperative interval, which was log-transformed due to its wide distribution (3-296 months). In addition, supplementary analyses were performed to examine the association between cognitive outcomes and time since radiotherapy within the subgroup of glioma patients who received radiotherapy. Lesion laterality and lesion location were not included in the primary analyses to avoid overfitting, given the limited sample size.

Given the high intercorrelation among white matter tracts, stepwise multiple regression was used as an exploratory approach to identify tract-level disconnection metrics associated with MMSE and JART scores. Finally, regression models were used to examine the relationship between CRI-leisure, white matter disconnection, and cognitive performance. All analyses were performed using JMP (version 18), with statistical significance set at *P *< .05 (2-tailed).

## Results

### Patient Characteristics

**Figure 1.**
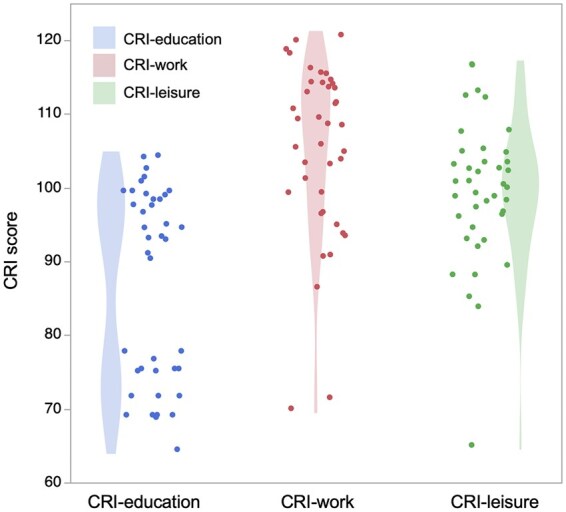
Distribution of Cognitive Reserve Index (CRI) subdomain scores. Violin plots illustrate the distribution of Cognitive Reserve Index (CRI) scores for each subdomain (education, work, and leisure), with individual participant data points overlaid. The width of each violin reflects the kernel density of the data. Individual dots represent single participants.

The summary of patient characteristics is presented in Table 1. Distributions of CRI subdomain scores are shown in Figure 1. Overall, 40 patients were included in the study, with a mean age of 52.7 years (range, 21-81), comprising 22 males and 18 females. Tumor laterality was left hemisphere in 15 patients, right hemisphere in 24, and bilateral in 1. Regarding tumor location, 19, 11, 4, and 6 tumors were located in the frontal lobe, temporal lobe, parietal lobe, and other regions, respectively. According to the WHO classification, 4, 16, 8, and 10 tumors were grades 1, 2, 3, and 4, respectively, while 2 were metastatic tumors. Eighteen patients had received adjuvant radiation therapy. All patients were evaluated in the chronic postoperative phase, at least 3 months after surgery. Although the timing of assessment varied across patients, all were clinically stable at the time of cognitive testing. Individual data on cognitive outcomes, pathological diagnoses, radiotherapy history, and timing of postoperative assessment are provided in Supplementary Table S1.

**Table 1.** Patient characteristics (*n* = 40)

	No. of patients
**Age years (mean ± SD)**	52.7 ± 13.8
**Sex (male:female)**	22:18
**Laterality (left:right:both)**	15:24:1
**Location**	
Frontal	19
Temporal	11
Parietal	4
Others	6
**KPS score (%)**	
100	18
90	13
80	6
70	3
**WHO grade**	
1	4
2	16
3	8
4	10
Metastasis	2
**Radiation therapy, *n* (%)**	18 (45%)

“Both” indicates bilateral or midline-crossing lesions. Metastatic tumors were not assigned a WHO grade. Others include insular, occipital, and deep-seated lesions. KPS, Karnofsky Performance Status. SD indicates standard deviation.

### Associations between CR, Patient Characteristics, and Cognitive Outcomes

**Figure 2.**
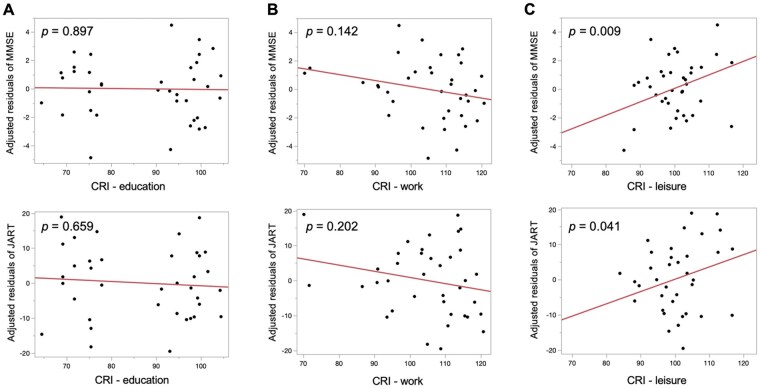
Associations between cognitive reserve subdomains and cognitive outcomes after covariate adjustment. Scatter plots show the relationships between CRI-education (A), CRI-work (B), and CRI-leisure (C) and adjusted residuals of MMSE (top) and JART (bottom), after adjustment for age, sex, Karnofsky Performance Status, WHO tumor grade, history of radiotherapy, and postoperative interval.

The summary results of the neuropsychological assessments and CRI scores are presented in Table 2 and Supplementary Figure S2. After adjustment for age, sex, KPS, WHO tumor grade, history of radiotherapy, and postoperative interval, MMSE and JART remained significantly correlated. Specifically, a significant positive association was observed between the adjusted residuals of MMSE and JART (*β* = 0.095, *r *= 0.48, *P *= .003) (Supplementary Figure S3). In unadjusted analyses, among the CRI subdomains, only CRI-leisure showed statistically significant correlations with both MMSE (*r* = 0.57, *P *< .001) and JART (*r *= 0.35, *P *= .034), whereas CRI-education and CRI-work did not show significant associations with either cognitive measure. To account for potential confounding, associations between CRI subdomains and cognitive outcomes were further examined using adjusted residuals after controlling for age, sex, KPS, World Health Organization (WHO) tumor grade, history of radiotherapy, and postoperative interval. After covariate adjustment, CRI-leisure remained significantly associated with both MMSE (*P *= .009) and JART (*P *= .041), whereas no significant associations were observed for CRI-education or CRI-work (Figure 2).

**Table 2.** Summary of neuropsychological assessment and cognitive reserve scores

Examination	Mean ± SD
**MMSE**	27.3 ± 3.2
**JART**	99.9 ± 11.2
**CRI-total**	97.9 ± 8.0
CRI-education	87.0 ± 13.1
CRI-work	105.2 ± 12.0
CRI-leisure	99.5 ± 9.6

Values are presented as mean scores with ranges. CRI-total represents the composite score derived from the education, work, and leisure subdomains. SD indicates standard deviation.

Abbreviations: CRI, Cognitive Reserve Index; JART, Japanese Adult Reading Test; MMSE, Mini-Mental State Examination.

Next, considering the biological characteristics of the tumors, we excluded 2 cases of intraventricular tumors (meningioma and schwannoma) and repeated the analyses. Consistent with the main findings, CRI-leisure remained significantly associated with both MMSE (*P *= .008) and JART (*P *= .029), whereas CRI-education and CRI-work again showed no significant associations. Furthermore, in analyses restricted to glioma cases, only CRI-leisure showed significant associations with MMSE (*P *= .0142) and JART (*P *= .0498).

Finally, in the analysis of patient background factors, MMSE but not JART was associated with sex (*P *= .008), KPS category (*P *= .032), and WHO tumor grade (*P *= .015). In analyses restricted to glioma cases, univariate comparisons showed that neither MMSE (*P *= .9514) nor JART (*P *= .7884) was associated with IDH mutation status. In analyses restricted to IDH-mutant glioma cases, no significant differences in MMSE or JART scores were observed between astrocytoma and oligodendroglioma. In contrast, a history of radiotherapy was significantly associated with lower MMSE scores (*P *= .0187), whereas no significant difference was observed for JART (*P *= .8574). Furthermore, among the 14 glioma patients who underwent radiotherapy, neither MMSE (*P *= .7835) nor JART (*P *= .1732) showed a significant correlation with the time interval since radiotherapy.

### Associations between White Matter Disconnection and Cognitive Performance

**Figure 3.**
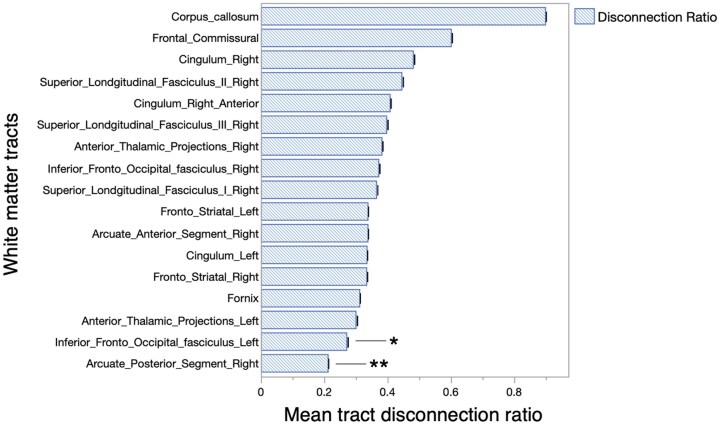
Mean disconnection ratios of white matter tracts across patients. The figure displays the 15 white matter tracts with the highest mean disconnection rates across the cohort, together with tracts whose disconnection rates were significantly associated with JART performance after covariate adjustment. Horizontal bars indicate the mean disconnection rate for each tract, and box plots represent the median and interquartile range, with whiskers indicating the range of observed values. Tracts are ordered from highest to lowest mean disconnection rate. Tracts showing significant associations with JART performance are marked (*P < .05, **P < .01 after covariate adjustment).

An overlap map of resection cavity ROIs from all patients is presented in Supplementary Figure S4. Disconnection analysis revealed that the most frequently disrupted tracts due to tumor resection included the corpus callosum, anterior commissure, right cingulum bundle, and right dorsal SLF (Figure 3 and Supplementary Figure S5). In contrast, among the 68 white matter tracts examined, multivariable regression analyses identified 2 tracts—the left IFOF and the right posterior SLF—as being significantly associated with lower JART scores (*P *= .023 and *P *= .004, respectively), after adjustment for clinical covariates. No significant associations were observed between white matter tract disconnection and MMSE scores. These associations remained statistically significant after covariate adjustment.

These associations remained statistically significant after covariate adjustment. Notably, the associations between these tracts and JART scores remained evident after excluding 2 cases of extra-axial tumors (left IFOF, *P *= .0056; right posterior SLF, *P *= .0152). However, in analyses restricted to glioma cases, these associations were no longer statistically significant.

**Figure 4.**
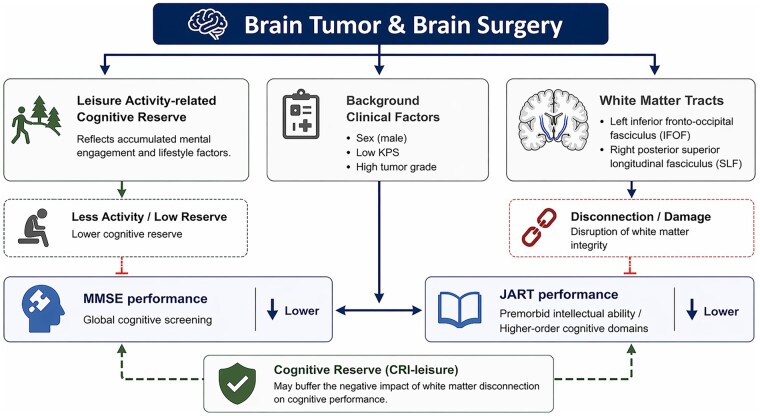
Conceptual framework of cognitive reserve and white matter disconnection in brain tumor patients. Brain tumor surgery may affect cognitive outcomes through background clinical factors and white matter tract disconnection. Disconnection or damage involving key white matter tracts, including the left inferior fronto-occipital fasciculus (IFOF) and right posterior superior longitudinal fasciculus (SLF), is associated with lower Japanese Adult Reading Test (JART) performance. Reduced leisure activity-related cognitive reserve may contribute to lower cognitive reserve and poorer Mini-Mental State Examination (MMSE) performance, whereas Cognitive Reserve Index-leisure (CRI-leisure) may buffer the negative impact of white matter disconnection on cognitive performance. MMSE and JART performance are shown as partially related but distinct cognitive outcomes.

The results are illustrated in Figure 4, which schematically summarizes the observed relationships between cognitive reserve, white matter disconnection, and neuropsychological outcomes in patients with brain tumors.

## Discussion

This study provides evidence that cognitive reserve, particularly leisure-related cognitive reserve, is associated with cognitive outcomes following brain tumor resection. Our findings align with prior literature highlighting the role of lifestyle factors in mitigating cognitive decline in neurodegenerative diseases^1^^,^^3^ and extend these principles to the postoperative brain tumor population. Notably, CRI-leisure showed an association distinct from the education and occupational subdomains with both MMSE and JART, highlighting the potential relevance of cognitively engaging leisure activities in supporting cognitive resilience.

Previous studies have primarily examined premorbid IQ and educational attainment as proxies for cognitive reserve, emphasizing their protective effects on cognitive function in neurological disorders; although studies specifically addressing cognitive reserve in patients with brain tumors remain limited, these investigations provide a useful framework for interpreting the present findings. Ownsworth et al^19^ reported that higher premorbid IQ was associated with greater psychological well-being and quality of life, even when cognitive functioning was reduced. MacPherson et al^20^^,^^21^ demonstrated that premorbid IQ predicted executive function and naming performance in patients with frontal lobe lesions, including stroke and brain tumors. Similarly, Campanella et al^22^ showed that premorbid IQ was the strongest predictor of preoperative language function and mitigated the effects of lesion volume in patients with brain tumors. Collectively, these findings are consistent with a role of cognitive reserve, as operationalized by premorbid IQ and related proxies, in mitigating the cognitive consequences of tumor- and surgery-related brain injury. The present study extends this literature by demonstrating that leisure-based CR, rather than education or occupation, plays a particularly important role in postoperative cognitive resilience.

The association of postoperative cognitive impairment with disruption of the left IFOF and right posterior SLF is consistent with prior evidence implicating these pathways in semantic processing, attention, and memory integration.^11^^,^^23^ Their apparent vulnerability to surgical disruption, together with their strong association with JART scores, emphasizes the importance of tract integrity in maintaining complex cognition. In Alzheimer’s disease cohorts, JART and the National Adult Reading Test (NART) are commonly used as indicators of premorbid intellectual ability and are closely related to higher-order cognitive functions; declines in these measures reflect current cognitive status and disease severity.^24^^,^^25^ Accordingly, reading-based premorbid IQ indices are not fully independent of current cognitive state, particularly in attention and language domains—a consideration that is likely relevant in patients with brain tumors. An important consideration in interpreting these findings is the limited sensitivity of the MMSE in the present cohort. MMSE scores showed a pronounced ceiling effect, with a substantial proportion of patients scoring near the maximum, which likely reduced the ability of this measure to detect subtle differences related to white matter tract disconnection or cognitive reserve.^26^ This limitation may partly explain why tract-specific associations were observed for JART but not for MMSE.

Anatomically, the IFOF and posterior SLF are major association tracts demonstrated by fiber dissection and tractography. Beyond their domain-specific roles, recent work has emphasized that the IFOF supports language, motor cognition, attention, and emotion, while more fundamentally contributing to cognitive control through context-dependent modulation of visual information.^27^ The IFOF links inferior/middle frontal gyri with the occipital extrastriate and basal temporal regions,^23^^,^^28^ whereas the posterior SLF connects the posterior middle temporal gyrus to the angular and, in part, the supramarginal gyrus.^11^ Although the left IFOF’s role, spanning semantic processing to integrative language, is relatively well established, the function of the right posterior SLF remains less clearly defined. Notably, the right sagittal stratum region traversed by the right posterior SLF corresponds to the temporo-parietal junction (TPJ), a hub for multisensory integration and higher-order processing.^29^ Intraoperative stimulation of the right posterior SLF can elicit spatial neglect, vertigo, and anomia,^29^ and our previous research has shown that glioblastomas involving the right TPJ and associated tracts markedly worsen postoperative quality of life by disrupting networks subserving attention, visuospatial cognition, emotion recognition, and visual fields.^30^ In our tract-wise analyses, disconnection of the right posterior SLF was consistently associated with poorer cognitive performance, even after accounting for clinical covariates. Because the posterior SLF is a major structural pathway connecting temporoparietal association cortex, including the TPJ, with distributed frontoparietal networks implicated in attention and cognitive control, this finding is consistent with the interpretation that the integrity of a right TPJ–posterior SLF network may be relevant to cognitive resilience.

In this study, leisure-related cognitive reserve was associated with cognitive outcomes in the context of white matter disruption, highlighting interindividual variability in postoperative cognitive trajectories. Rather than implying a direct compensatory interaction or deterministic effects, these findings are consistent with conceptual models of cognitive reserve in which greater reserve is associated with more flexible or efficient utilization of preserved neural resources.^4^ Importantly, our results do not support the use of leisure-related cognitive reserve as a determinant of surgical decision-making, such as defining permissible resection extent. Instead, they suggest that cognitive reserve may be informative for understanding individual vulnerability to postoperative cognitive impairment. From a preventive and supportive perspective, engagement in cognitively stimulating leisure activities may represent a potentially modifiable factor relevant to long-term cognitive resilience in patients undergoing brain tumor treatment. Consistent with this view, prior work has suggested that cognitive and experiential factors can influence functional outcomes following structural brain injury.^31^ Together, these observations support a framework in which cognitive reserve contributes to heterogeneity in postoperative cognitive outcomes, without implying direct guidance for surgical planning.

The leisure-related component of the CRI captures engagement in cognitively stimulating activities across the lifespan, such as reading, playing musical instruments, engaging in hobbies, and participating in social and cultural activities. Unlike education or occupational attainment, this component reflects ongoing and potentially modifiable cognitive engagement, which may help contextualize its association with postoperative cognitive outcomes observed in this study. Beyond showing tract-level effects, this study revealed that among CRI domains, only leisure significantly influenced prognosis in patients with brain tumors. Leisure activities—diverse pursuits undertaken outside occupational and familial obligations—promote well-being, alleviate stress, and support mental health through relaxation, skill acquisition, and social interaction.^32^^,^^33^ These effects are underpinned by resilience, the capacity to adapt to adversity, which leisure strengthens via intrapersonal and interpersonal pathways.^34^ Participation in various leisure activities can reduce depressive symptoms through resilience and protect against stress even during large-scale disasters.^35^^,^^36^ In patients with brain tumors, where localized structural damage is salient, the mechanisms by which leisure-related CR operates may differ and merit targeted study. In contrast, the lack of significant associations for the education and occupational subdomains may reflect the relatively limited variability in educational attainment within the Japanese population, as well as the potential influence of disease-related functional decline or retirement on occupational status. By comparison, leisure activities are more flexible, ongoing, and more likely to reflect active cognitive engagement, which may account for their stronger association with postoperative cognitive resilience.

Taken together, these findings indicate that individual differences in cognitive reserve—particularly those related to leisure activities—are associated with variability in cognitive vulnerability following focal white matter disconnection caused by brain tumor surgery. Rather than implying direct clinical decision-making rules, this study provides a conceptual framework for understanding why patients with similar structural brain injuries may experience markedly different cognitive outcomes.^37^^,^^38^ Although the cohort was heterogeneous with respect to tumor pathology and location, this was an intentional design choice. The primary aim of this study was not to compare tumor subtypes but to examine a general principle: the relationship between structural white matter disconnection and cognitive reserve across patients with brain tumors. This heterogeneity may enhance the generalizability of the findings.

Several limitations should be acknowledged. First, the sample size was modest, limiting statistical power for subgroup analyses based on tumor pathology or location. The tract-level disconnection analyses should be interpreted as exploratory, given the relatively large number of statistical tests performed in a small and heterogeneous cohort, which may increase the risk of type I error. Accordingly, we focused on consistent patterns across analyses rather than isolated significant findings and avoided overinterpretation of marginal results. Given these constraints, the present study was primarily powered to detect relatively large effect sizes, and smaller but potentially meaningful associations may not have been identified. While restricting the analysis to glioma patients would reduce biological heterogeneity, the present study included a relatively broad clinical population. Additional subgroup analyses showed generally consistent patterns, although statistical significance was attenuated due to reduced sample size. Moreover, while the overall cohort size was sufficient for the primary analyses, it may have been insufficient to detect tract-level associations in voxelwise structural disconnection mapping, especially in subgroup analyses, given the influence of resection cavity extent and the requirement for adequate statistical power. Second, the cross-sectional design precludes causal inference regarding the protective role of CR, and reverse causality cannot be excluded. Third, CR was assessed by self-report, which may be influenced by postoperative cognitive changes despite its conceptualization as a premorbid construct. Fourth, the absence of preoperative cognitive testing and diffusion MRI limits direct comparison of pre- and postoperative network integrity. Finally, the MMSE, while useful as a global cognitive screening tool, does not provide a detailed neurocognitive profile, which should be considered when interpreting the present findings. Despite these limitations, we provide novel evidence that cognitive reserve, particularly leisure-based cognitive reserve, is associated with variability in cognitive outcomes in the context of tract-level disconnection in patients with brain tumors. These findings suggest that individualized assessment of cognitive reserve may complement anatomical information when interpreting postoperative cognitive trajectories and planning supportive care.

## Conclusion

Our findings highlight the relevance of cognitive reserve, particularly leisure-related cognitive reserve, in relation to cognitive outcomes after brain tumor surgery. Even in the presence of structural white matter disconnection, higher CRI-leisure scores were associated with relatively preserved cognitive performance. Disruption of the left IFOF and right posterior SLF was associated with poorer cognitive performance, with the strength of these associations varying according to individual reserve capacity. Together, these findings suggest that cognitive reserve may contribute to interindividual variability in postoperative cognitive outcomes and underscore the need for further investigation in larger, prospective cohorts.

## Supplementary Material

vdag175_Supplementary_Data

## Data Availability

The datasets generated and/or analyzed during the current study are not publicly available due to ethical and privacy restrictions involving human participants, but are available from the corresponding author on reasonable request.
