# Supplementary material for: Leisure-related cognitive reserve and postoperative cognitive outcomes after brain tumor surgery
Source: Neurooncol Adv. 2026 Jul 27;8(1):vdag175. doi: 10.1093/noajnl/vdag175 (PMC13424432; doi:10.1093/noajnl/vdag175)
Supplement: vdag175_Supplementary_Data [file vdag175_supplementary_data.docx]

**Supplemental Figure S1. Workflow of disconnection analysis**
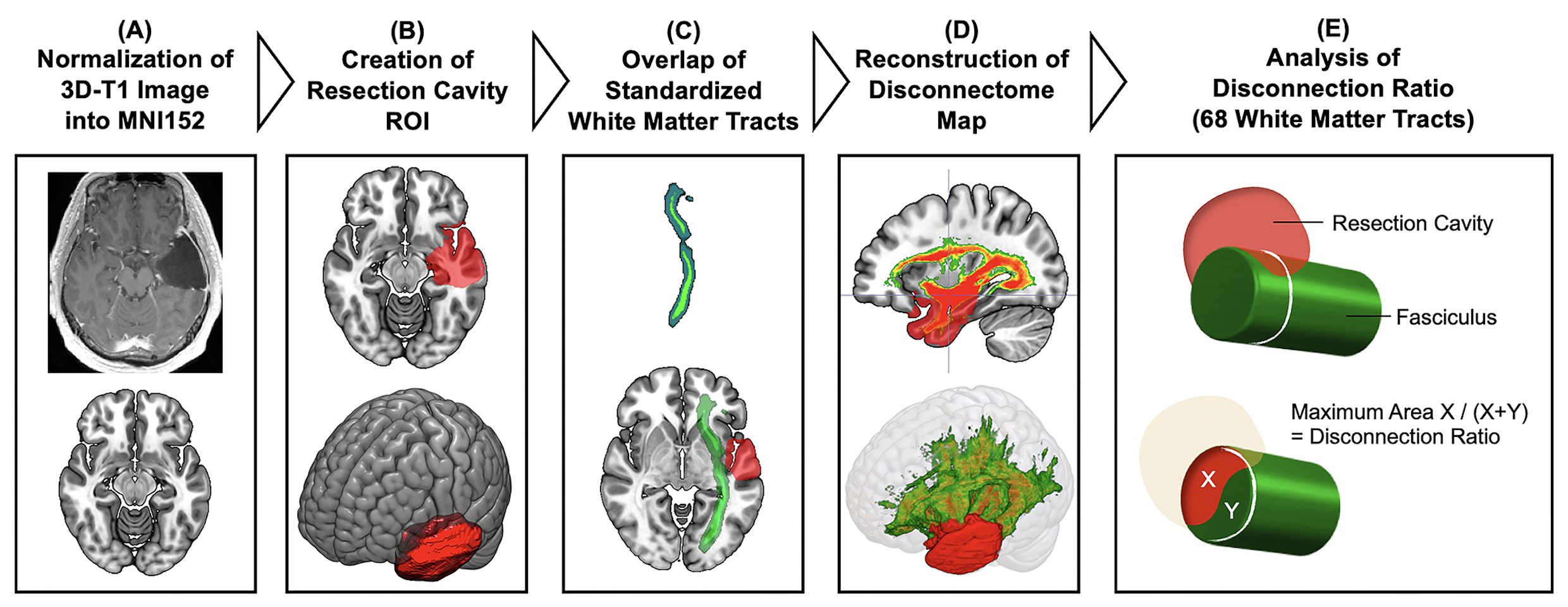


After standard preprocessing of postoperative T1-weighted images (A), a region of interest (ROI) of the resection cavity was created (B). On the BCBtoolkit and Tractotron software platform, each standard white matter tract was overlaid (C), and a disconnection map of white matter networks intersecting with the resection cavity ROI was generated (D). Finally, the maximum disconnection ratio was calculated across all 68 white matter tracts (E).

**Supplemental Table S1. Cognitive outcomes, pathological diagnoses, radiotherapy history, and timing of postoperative assessment in individual cases (N = 40)**


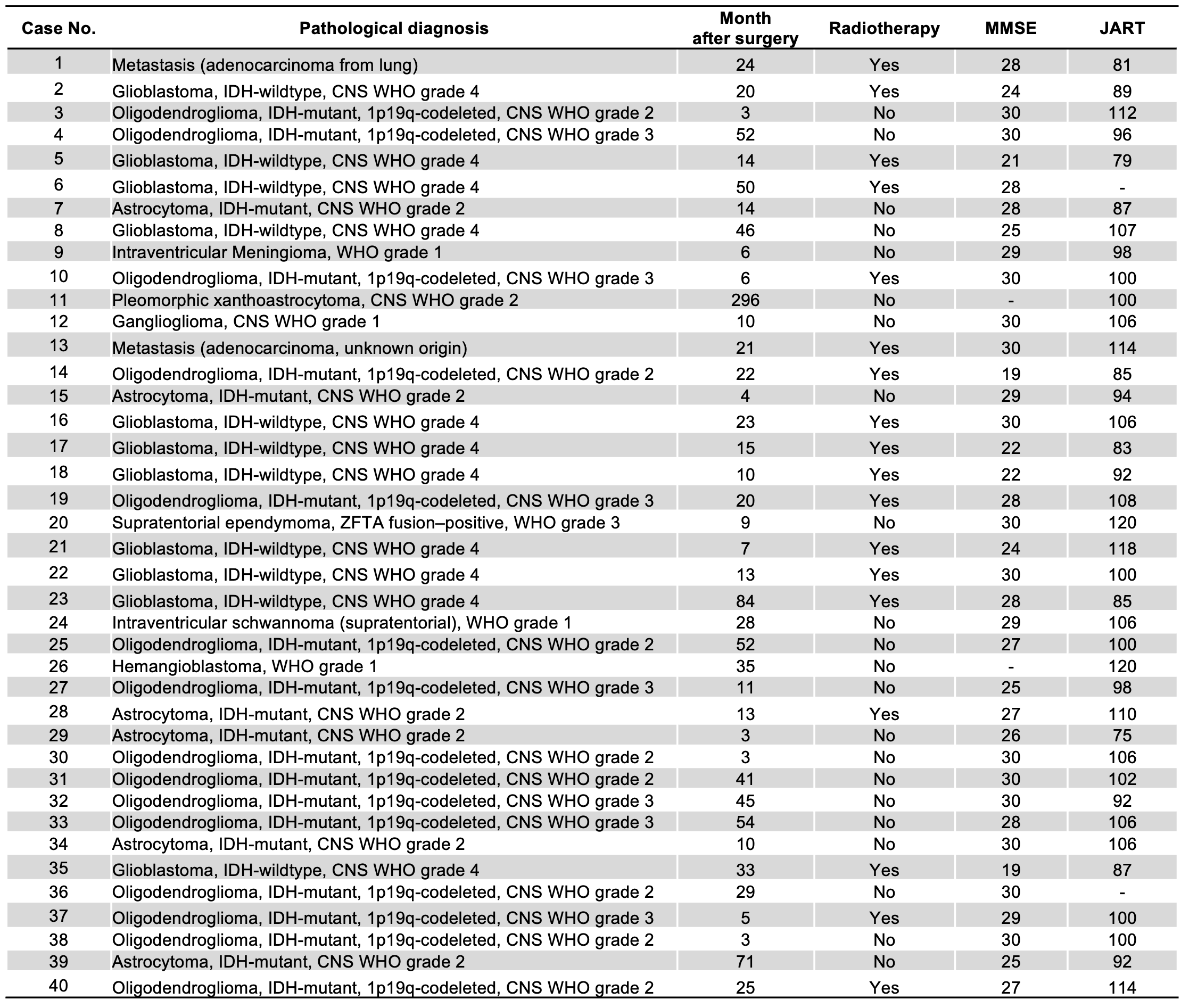


Missing values indicate that the corresponding neuropsychological test could not be completed or was unavailable at the time of assessment. MMSE, Mini-Mental State Examination; JART, Japanese Adult Reading Test.

**Supplementary Figure S2. Distribution of cognitive scores in the cohort**


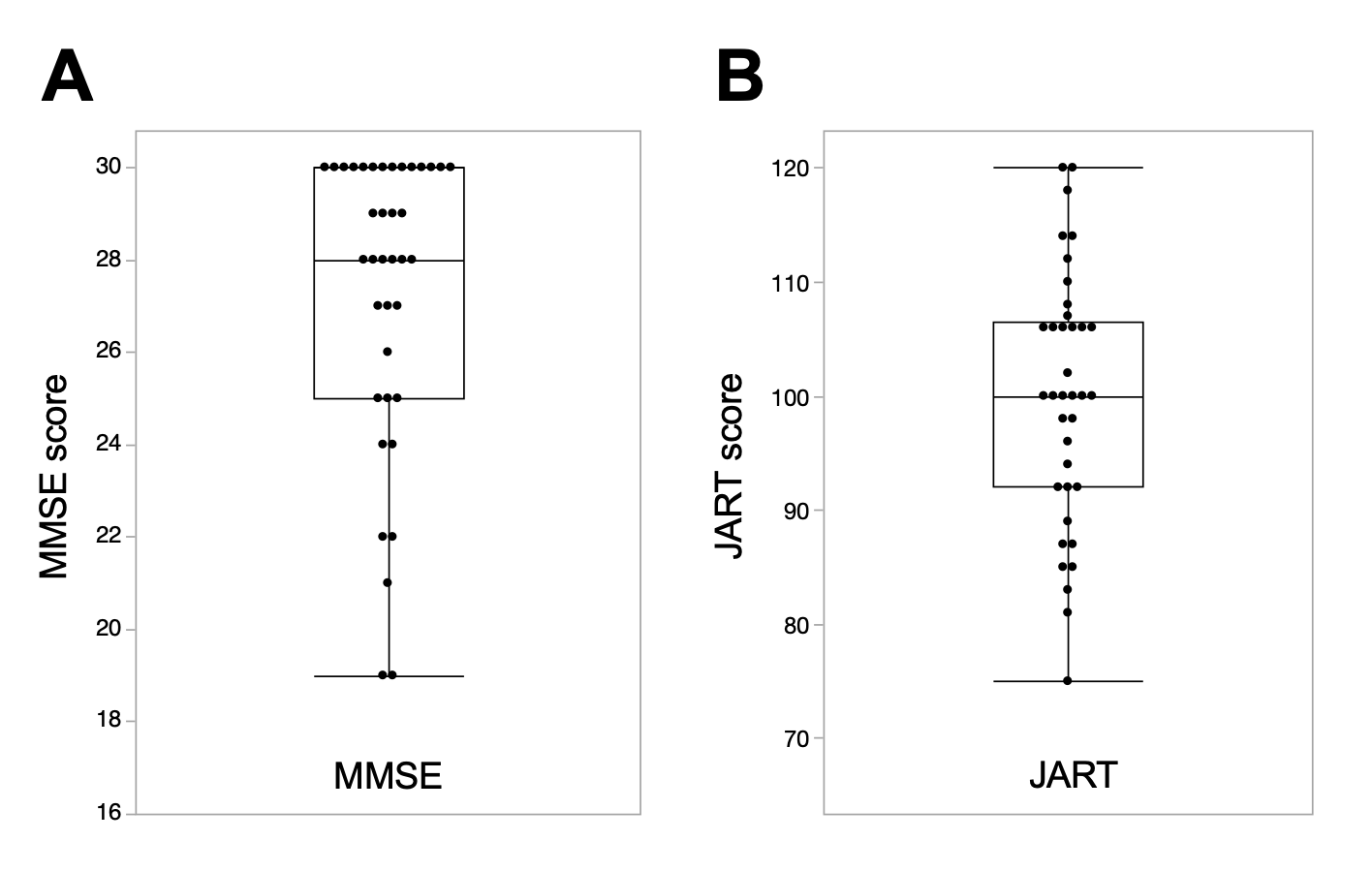


(A) MMSE scores show a pronounced ceiling effect, with many patients scoring near the maximum.

(B) JART scores show a wider distribution without a marked ceiling effect.

MMSE, Mini-Mental State Examination; JART, Japanese Adult Reading Test.

**Supplementary Figure S3. Association between adjusted residuals of JART and MMSE**


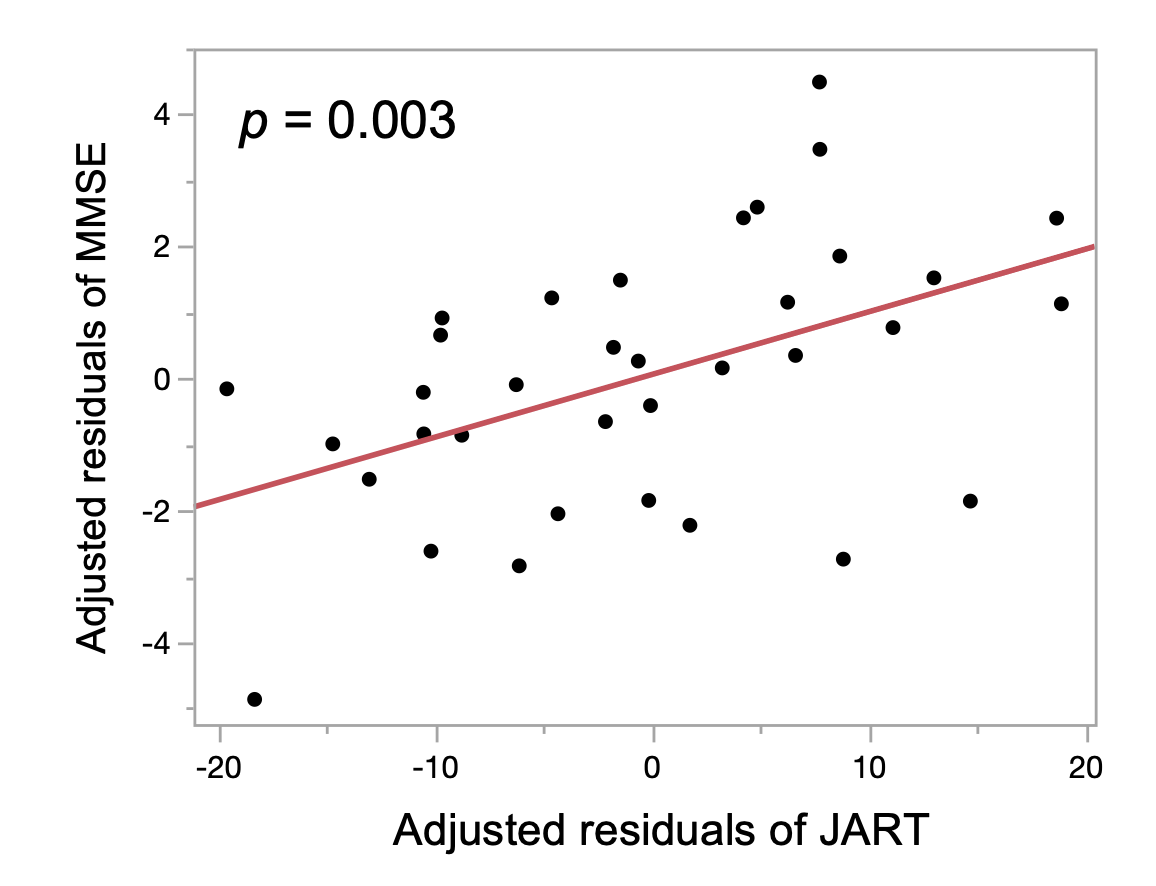


Scatter plot showing the relationship between adjusted residuals of JART (x-axis) and adjusted residuals of MMSE (y-axis) after controlling for age, sex, Karnofsky Performance Status, WHO tumor grade, history of radiotherapy, and postoperative interval. The red line represents the fitted linear regression line. A significant positive correlation was observed (r = 0.48, p = 0.003).
MMSE, Mini-Mental State Examination; JART, Japanese Adult Reading Test.

**Supplemental Figure S4. Overlap map of tumor lesions (N=40)**

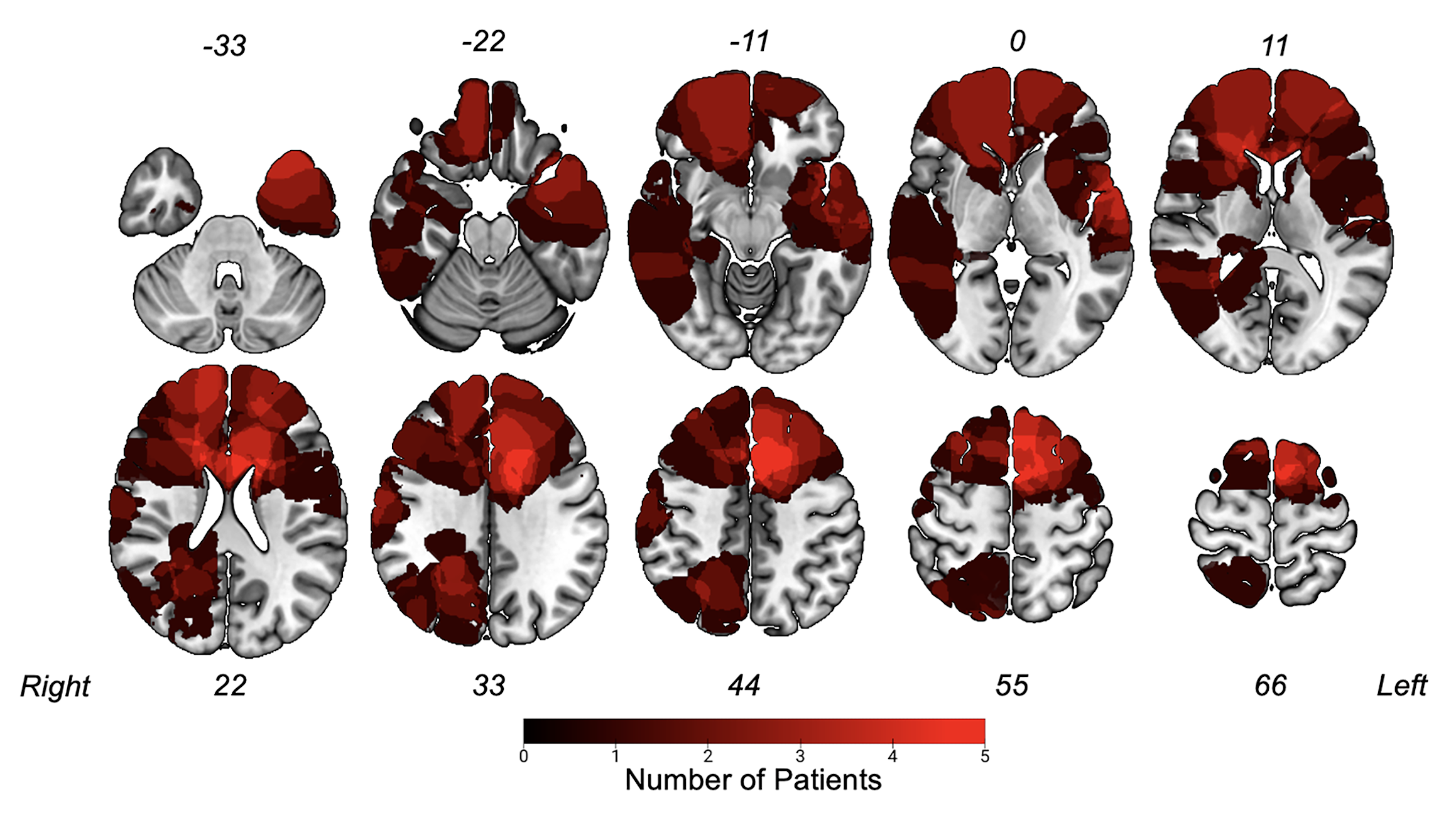


Red regions indicate the areas of greatest overlap in our case group (N = 5). Numbers at the top and bottom of the slices represent the coordinates of the MNI template. MNI, Montreal Neurological Institute.

**Supplementary Figure S5. Mean tract disconnection ratio across patients**


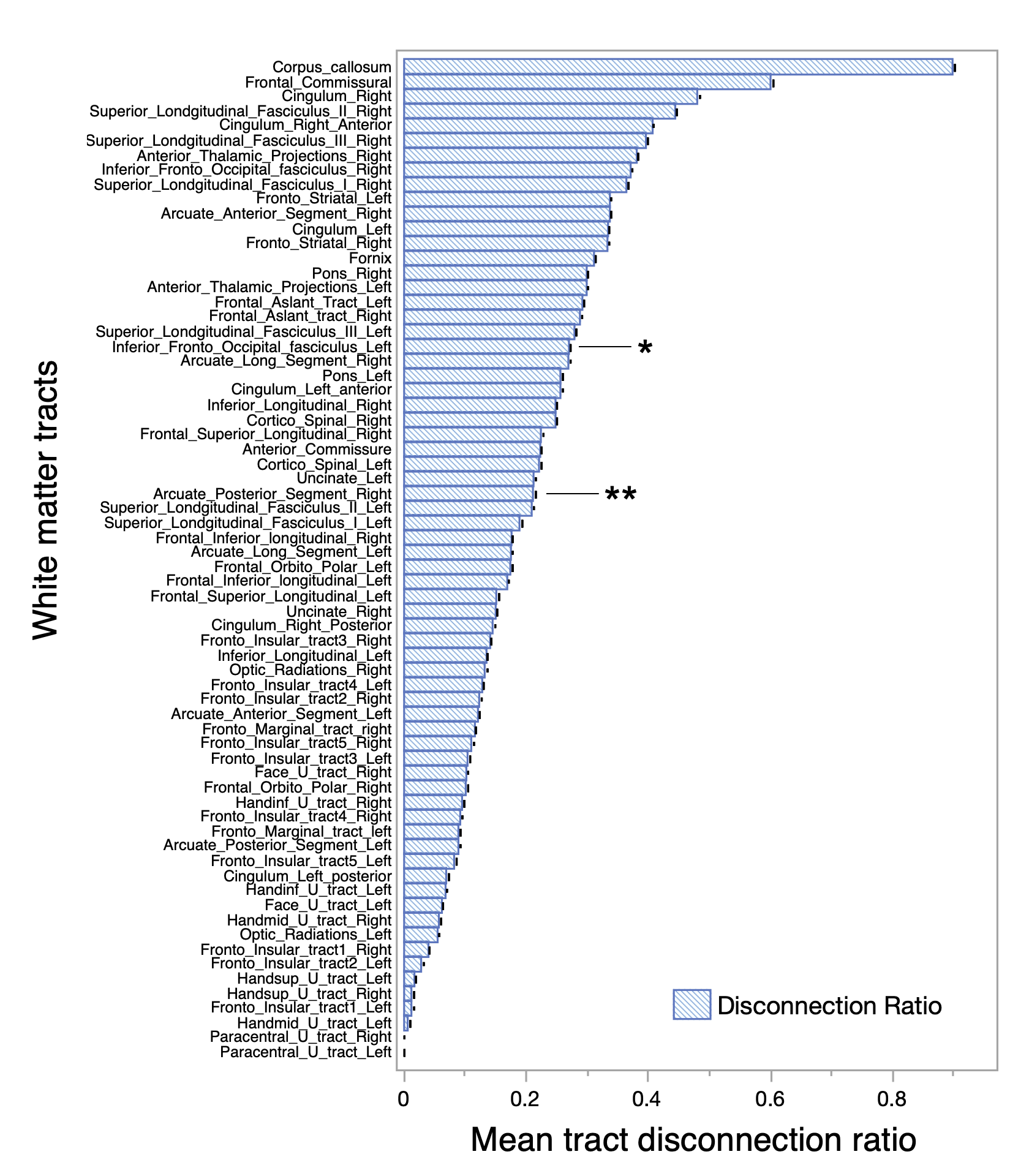
The figure displays the disconnection rates for all examined white matter tracts. Horizontal bars indicate the mean disconnection rate for each tract, and box plots represent the median and interquartile range, with whiskers indicating the range of observed values. Tracts showing significant associations with JART performance after covariate adjustment are indicated (* p < 0.05, ** p < 0.01). This figure provides the full tract-wise overview corresponding to the selected tracts shown in the main figure.
